# A Longitudinal Study of Relationships between Identity Continuity and Anxiety Following Brain Injury

**DOI:** 10.3389/fpsyg.2017.00648

**Published:** 2017-05-12

**Authors:** R. S. Walsh, Orla T. Muldoon, Donal G. Fortune, Stephen Gallagher

**Affiliations:** ^1^Department of Psychology, Manchester Metropolitan UniversityManchester, UK; ^2^Department of Psychology, University of LimerickLimerick, Ireland; ^3^Health Service ExecutiveCork, Ireland

**Keywords:** brain injury, social identity, self-categorisation, identity continuity, anxiety

## Abstract

**Objective:** Anxiety is of particular importance following acquired brain injury (ABI), because anxiety has been identified as a significant predictor of functional outcomes. Continuity of self has been linked to post ABI adjustment and research has linked self-discrepancy to anxiety. This longitudinal study investigates the impact of affiliative and ‘self as doer’ self-categorisations anxiety.

**Materials and Methods:** Data was collected at two time points. Fifty-three adult ABI survivors participating in post-acute community neuro-rehabilitation participated at time one and 32 of these participated at time two. Participants completed a 28-item identity questionnaire based on Leach et al.’s (2008) multicomponent model of ingroup identification which measured the strength of affiliative and self as doer identities. Anxiety was measured using the Hospital Anxiety and Depression Scale.

**Results:** Analysis indicates a significant mediated relationship between affiliative identification and anxiety via self as doer identification. Contrary to initial prediction, this relationship was significant for those with consistency in affiliative self-categorisation and inconsistency in ‘self as doer’ self-categorisation.

**Conclusion:** These findings can be interpreted as evidencing the importance of identity continuity and multiplicity following ABI and contribute to the understanding of these through the use of a social identity approach.

## Introduction

Acquired brain injury (ABI) is one of the most prevalent neurological impairments, affecting approximately 1 in 500 people globally, with a particular impact on young children, young adults, and those over 65 (Jones et al., 2011). In the aftermath of ABI anxiety is of particular significance because anxiety has been identified as a significant predictor of functional outcome in brain injury survivors (Ponsford et al., 2008). In this context an individual’s perceptions of ‘Continuity of self,’ a concept originating in the nursing literature (Secrest and Zeller, 2003) is crucial to well-being (Haslam et al., 2008). Continuity of self can be understood as the experience of oneself in terms of one’s connection with others, and one’s wholeness or integrity of being. On the other hand, discontinuity of self can be thought of in terms of dependence, disconnection, and threatened integrity (Secrest and Zeller, 2003).

The concept of continuity of self is one which has been usefully applied in a relational approach to rehabilitation (Bowen et al., 2010). Extending this line of thought, the concept of self-continuity may provide a focal point where meaning, belonging, and doing can be understood as coming together. Furthermore, this coming together of meaning, belonging, and doing may be fundamental to mental health, specifically anxiety, following ABI (Bowen et al., 2010). Previous research has shown that higher levels of pre to post injury self-discrepancy (i.e., ‘who I am now’ vs. ‘who I was before my injury’) are associated with higher levels of anxiety in survivors of traumatic brain injuries (Cantor et al., 2005). In contrast to self-discrepancy, continuity of self, defined as the experience of oneself as continuous with ‘who I was before’ (Secrest and Zeller, 2003, p. 244) has been linked to adjustment following traumatic brain injury (Bowen et al., 2010). The research presented in this paper applies a specific framework for understanding identity and selfhood, that of the social identity approach, to a longitudinal investigation of relationships between self-categorisation, continuity of self, and anxiety following ABI.

The social identity approach is a psychological metatheory incorporating social identity theory (SIT) and self-categorisation theory (SCT; Haslam et al., 2011). It is an approach particularly well-suited to application in the study of ABI because it focuses on the ‘we’s’ that individuals ascribe to. Moreover, it focuses on how it is that when individuals self-categorize as members of a particular group they understand themselves as part of a ‘we’ that interacts with ‘others.’ Social identity was defined by Tajfel (1982) as a person’s knowledge that they belong to given social groups and that membership of these groups has emotional and value significance for them. SCT has a focus on the shift behind individuals’ self-categorisation as distinct individuals to self-categorisation as members of collective groups. SCT is focused on how ‘we’ behave and how ‘others’ behave toward ‘us’ as a consequence of our group memberships, of our social identities (Walsh et al., 2012).

The social identity approach holds a unique perspective. Whereas analysis generally begins with the ‘the individual in the group,’ social identity analysis begins with the group in the individual (Reicher et al., 2010). Building on this perspective the present study investigates how social identities, evidenced by participants self-categorisations might impact anxiety following ABI. In particular, it will build on previous research that distinguishes between self-categorisations based on belonging (affiliative identities; e.g., family and national identities) and self-categorisations based on participation in meaningful activity (self as doer identities; e.g., occupational and sporting identities – ‘I am a farmer,’ ‘I am a golfer’; Walsh et al., 2014). Self-categorisation refers to the individual self-assignations that people make based on the social groups that they belong to. Self-categorisations matter because they provide the psychological basis for identification and its consequences,^1^ including for example symptom appraisal and response, health related norms and behaviors and clinical outcomes (Haslam et al., 2009). In the majority of contexts, identities structure what people do, what they think and what they can achieve (Haslam, 2014).

Given we understand that membership of social groups is a fundamental base on which individuals construct their sense of self, it is not a person’s nominal group memberships that need to be considered by researchers but their subjective self-categorisations (Haslam, 2014). Meaning is fundamental. As such researchers, and clinicians need to attend to how people understand themselves. In order to achieve this we followed the advice of Ashmore et al. (2004). Ashmore et al. (2004) highlight the importance of self-categorisation and advise researchers to provide respondents with the opportunity to answer open ended questions about their group memberships. The approach advocated by Ashmore et al. (2004, p. 86) ensures that participants are referring to the phenomenologically ‘correct’ social category in their responses.

Secrest and Zeller (2003) report that identity discontinuity in stroke survivors was experienced as a threat to integrity whereas continuity of self was associated with an experience of integrity of being. Tying this continuity of self to mental health, Sani et al. (2008) suggest that when individuals have a coherent, collective, self-understanding there are positive consequences for psychological well-being.

Therefore, given the associations between identity (dis)continuity and well-being, in this study we explore the impact of self-categorisation, and of the (in)consistency across time of these self-categorisations, in those affected by ABI. We also examine whether strength of identification with two distinct types of self-categorisations, those related to inclusion in social groups and those contingent on engagement with activity (Walsh et al., 2012), impacts on levels of anxiety.

Identity or ‘self’ can be understood as a dynamic process (Simon, 2004). For each of us, knowing who we are, where we have come from, and feeling an inner sense of personal and collective continuity across time is fundamental to our individual and collective well-being (Sani et al., 2008). Previous research into identity and ABI has looked at the discrepancy between pre and post injury selves. This line of inquiry indicates that brain injury is often accompanied by a feeling of discrepancy between ‘who I am now’ and ‘who I was before the injury’ (Cantor et al., 2005) that is in turn related to affective disorder. But what of consistency and change regarding self-categorisation in the post-injury self? Gracey et al. (2008) concluded that, in part at least, ABI survivors make sense of themselves in terms of the meanings attached to social activities that they engage in and that are significant to them after their injury. In a similar vein, Douglas (2012) reported that when ABI survivors engaged in meaningful social activity, this engagement supported social identity construction with knock on effects for affective well-being. Further, in previous cross-sectional research, a differential impact of the type of identifications arising from affiliative (or belonging) identities (such as family) and social identities that build around participation in meaningful vocational and leisure activities was observed in those recovering from ABI (Walsh et al., 2014).

In the context of ABI, relationships between self-categorized identifications, the consistency of these self-categorisations, and outcome have not, as yet, been investigated. We hypothesize that a key variable is whether or not people are consistent in their self-categorisations over time. Based on the existing literature, we hypothesized that consistency in self-categorisation would indicate identity continuity and be inversely related to anxiety. To illustrate this with a concrete example our suggestion was that those who consistently identified strongly with their families would have an identity base which would allow them to engage with meaningful activity. Further, in line with previous research we suggested that affiliative identification has a significant effect on anxiety via strength of self as doer identification and so in our example, strong family identification would effectively facilitate, for instance, attending and watching sport with family members which would in turn allow survivors to see themselves as active sports fans. In order to be significant, this relationship would also require consistency in affiliative self-categorisation. Finally, we expected that consistent identifications/self-categorisations based on engagement with meaningful activities would have a beneficial impact upon individual’s anxiety levels.

**FIGURE 1:**
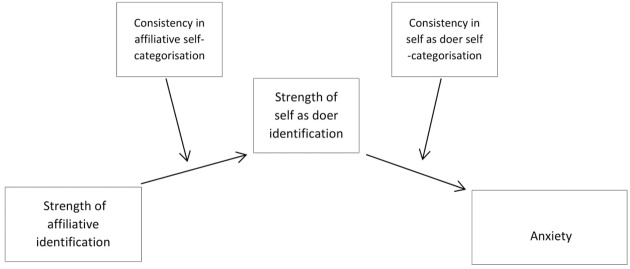
**Hypothetical model of relationships between identification, consistency, and anxiety**.

The hypothetical model is represented in **Figure 1** below.

## Materials and Methods

### Ethics Statement

This study was carried out in accordance with the recommendations of the University of Limerick Ethics Committee with written informed consent from all subjects. All subjects gave written informed consent in accordance with the Declaration of Helsinki. The protocol was approved by the University of Limerick Ethics Committee.

### Participants

Following receipt of the appropriate ethical approval, 53 adult survivors (39 men and 14 women) of brain injury, taking part in post-acute community neurorehabilitation with a national brain injury service provider in south west and mid-west Ireland took part in phase one of this study. Participants average age was 44 years (*SD* = 12.32). The youngest participant was 20 years and the oldest was 65 years. Average time since injury was 7 years (*SD* = 7.54; range: 1–27 years). Twenty-two participants had an ABI as a result of stroke. The other 31 participants had an ABI as a result of road traffic accidents (*n* = 15), falls (*n* = 7), tumor (*n* = 4), assault (*n* = 2), hypoxia (*n* = 2), and other (*n* = 1). One participant did not complete the affiliative identification component of the questionnaire and another participant did not complete the self as doer identification component of the questionnaire.

Thirty-two of those who took part in the first phase of this study took part in the second phase. Reasons that participants were not available for follow up included discharge from service, hospitalization and being on holiday. Participants were spread over a large geographical area (i.e., there was up to 125 miles between locations) and this meant that if participants were not available while we were in their area it was not always possible to meet with them. Twenty-five of those who participated at time two were men and seven were women. Fourteen had a brain injury as a result of stroke and 18 as a result of other causes^2^ (other causes: road traffic accident = 10; falls = 3; hypoxia = 2; assault = 1; unknown = 1). The mean age of participants was 44 years. The youngest participant was 20 and the oldest was 65. Mean time since injury was 6 years with the most recent injury being 1 year and the most distant being 27 years (SD time since injury = 5.73 years). Independent samples *t*-test were conducted to examine whether the demographics, in terms of age and time since injury, of those who participated at both times 1 (*n* = 21) and 2 (*n* = 32) differed significantly from the demographics of those who participated at time 1 only. There were no significant differences. The mean age of those who participated at time 1 only was 47.04 years (*SD* = 10.97). The mean age of those who participated at both times was 43.69 years [*SD* = 12.89; *t*(51) = 0.98, *p* = 0.33]. The mean time since injury of those who participated at time 1 only was 8.80 years (*SD* = 9.57), the mean time since injury of those who participated at both time points 1 and 2 was 5.94 years [*SD* = 5.73; *t*(50) = 1.35, *p* = 0.18]. Gender was not significantly different between those who participated at time 1 only and those who participated at both time points, *x^2^*(1, *N* = 53) = 0.31, *p* = 0.58. Cause of injury was not significantly different between these groups *x^2^*(1, *N* = 53) = 0.00, *p* = 0.95.

### Measures

#### Self-categorisation

Participants completed a 28-item identity questionnaire based on Leach et al.’s (2008) valid and reliable multicomponent model of ingroup identification. This instrument measured the strength of affiliative and self as doer identities. Self-categorized affiliative identity was established with the question ‘Which group of people you belong to is most important to who you are?’ Self-categorized self as doer identity was accessed via the open question ‘Which of the things you do is most important to who you are?’ (see Appendix 1 and Appendix 2 below).

#### Strength of Affiliative and Self as Doer Identification

The strength of identification measure comprises a 13 item questionnaire which measures group level self-investment (solidarity; satisfaction and centrality) and group level self-definition (individual self-stereotyping; ingroup homogeneity). In a slight change, question 7 of the original, ‘Being [in-group] gives me a good feeling,’ was left out because it appears to be very similar to another item ‘It is pleasant to be [in-group]’ and it was felt that this had the potential to cause confusion with ABI participants. A seven-point Likert scale was employed to ascertain individual item scores (1 = Agree; 7 = Disagree) as per Leach et al. (2010). All five subscales of the multicomponent model of in-group identification were summed to provide an overall measure of identity strength for both active and affiliative identity. The identity questionnaire first measured self as doer identity and then affiliative identity. Sample items include ‘I feel committed to...’; ‘The fact that I am....is an important part of my identity’; ‘I feel glad to be...’ Cronbach’s α for affiliative identity in-group identification = 0.81. Cronbach’s α for the self as doer in-group identification questionnaire = 0.82.

#### Consistency and Variability in Self-categorisation

Self-categorized affiliative and self as doer identities were established at time 1 and time 2. Where participants chose the same identity at time 1 and time 2 participants were coded as having self-categorisation consistency (code = 0). Where participants chose a different identity at time 1 and time 2 participants were coded as having self-categorisation inconsistency (code = 1). This procedure was followed for both affiliative and self as doer self-categorisations.

#### Anxiety

Anxiety was measured with the anxiety subscale of the Hospital Anxiety and Depression Scale (HADS; Zigmond and Snaith, 1983). The anxiety subscale scale contains seven four-point items, from 0 (not present) to 3 (considerable) designed to assess anxiety (e.g., ‘Worrying thoughts go through my mind’) with scores of 8 or more being indicative of caseness (Snaith, 2003; 8 of the 32 participants in this study scored 8 or over on HADSA). The HADS scale was designed in the setting of an outpatient clinic in a general medical hospital. Many studies have since confirmed the validity of the HADS scale and it has been shown to be an instrument suited to broad application (Snaith, 2003). In the present study a Cronbach’s α’s of 0.84 was obtained for the anxiety subscale.

### Procedure

Following receipt of ethical approval, data was collected across two time points with an average interval of 13 months between time 1 and time 2 data collection. The shortest individual interval between data collection time points was 8 months and the longest was 16 months (*SD* = 2.64). It was explained to all participants, at both time points, that participation was optional and that they were free to withdraw at any time.

Participants were interviewed in locations most convenient for them. Data was collected by two researchers, both of whom had police clearance to engage with vulnerable participants.

### Statistical Analyses

Hayes (2013) argues that establishing relationships between variables is an important part of scientific research. Hayes (2013) also cautions that even when a relationship between variables can be established, the identification of an association does not necessarily equate to understanding. One way to improve understanding is to consider questions regarding how and when one variable might impact another. ‘How’ is a question of underlying process and ‘when’ a question pertaining to the boundary conditions of a putative association. Mediation analysis is suited to the former and moderation the latter (Hayes, 2013).

The mean, standard deviation and reliability for all measures was calculated. Associations between strength of affiliative identification, strength of self as doer identification, anxiety, cause of injury, age, gender, and time since injury were investigated with correlation analysis (Pearson’s *r*). Lastly, PROCESS model 21 (Hayes, 2013), was employed to investigate whether there was a mediated relationship between strength of affiliative identification and anxiety via strength of self as doer identification that was moderated by stability of self-categorisations. Following the guidance offered by Hayes (2009) bootstrapping to 5000 was conducted.

## Results

### Preliminary Analyses

Analyses began by calculating the mean, standard deviation, and range of all variables. These along with *t*-test indicated that there was no significant difference between measures across time points are presented in **Table 1**.

**Table 1:** Means and standard deviations of all variables.

		T1 strength of affiliative identification	T1 strength of self as doer identification	T1 HADSA	T2 strength of affiliative identification	*t*-test T1 affiliative – T2 affiliative	T2 strength of self as doer identification	*t*-test T1 doer – T2 doer	T2 HADSA	*t*-test T1 HADSA – T2 HADSA
*N*	Valid	52	52	53	32		32		32	
Mean		82.56	75.65	6.08	82.41		78.69		5.53	
Standard deviation		9.11	12.37	4.5	8.54	*t* = 0.000	10.86	*t* = 0.123	4.17	*t* = 0.308
Minimum		57	44	0	62	*p* = 1.00	56	*p* = 0.90	0	*p* = 0.76
Maximum		91	91	17	91		91		19	

### Associations between Identities, Demographics, and Anxiety

Analysis next proceeded to examine relationships between variables using correlation analysis (Pearson’s *r*). Results indicated that strength of affiliative identification was significantly associated with strength of self as doer identification, and self as doer identification was significantly associated with anxiety. The demographic factors cause of injury (stroke/other) and gender (male/female) were significantly correlated with strength of affiliative identification and strength of self as doer identification respectively. Figures are presented in **Table 2**.

**Table 2:** Correlations between identification, anxiety and demographic variables (time 1 correlations are above the diagonal and time 2 correlations below).

		Affiliative	Self as doer	Anxiety	Cause of injury	Age	Gender	Time since injury
Strength of affiliative identification	-		0.54ˆ*	-0.04	-0.29ˆ*	0.16	0.08	-0.03
Strength of self as doer identification	-	0.50ˆ**		-0.32ˆ*	-0.19	0.19	-0.32ˆ*	-0.06
Anxiety	-	0.11	-0.36ˆ*		-0.03	-0.07	0.23	0.04
Cause of injury	-	-0.34ˆ^†^	-0.23	0.14		-0.58ˆ**	-0.19	0.29ˆ*
Age	-	0.15	0.13	-0.05	-0.73ˆ**		-0.11	0.14
Gender	-	12	-0.25	0.06	-0.14	0.13		-0.10
Time since injury	-	0.01	-0.09	0.14	0.27	0.03	-0.06	1

*^∗∗^Correlation is significant at the 0.01 level (two-tailed); ^∗^correlation is significant at the 0.05 level (two-tailed); ^†^correlation < 0.06.*

### Stability and Variability in Self-categorisation

Twenty-three participants (72%) self-categorized with the same affiliative identities at time points 1 and 2. Nine participants (28%) were inconsistent in their affiliative self-categorisation from T1 to T2. Fourteen participants (44%) self-categorized with the same self as doer identities at time points 1 and 2. Eighteen participants (56%) were inconsistent in their self as doer self-categorisations at each time point. The specific self-categorisations elicited from participants are presented in Appendix 1.

Correlation analysis indicated that for those participants whose self-categorized affiliative identities were consistent across time there was a significant correlation between strength of affiliative and self as doer identities: *r* = 0.647, *p* = 0.00, *N* = 23; for those whose affiliative identities were inconsistent there was no significant correlation between affiliative and active identities: *r* = -0.045, *p* = 0.909, *N* = 9.

Analysis indicated a significant correlation between strength of self as doer identification and anxiety, *r* = -0.66, *p* = 0.00, *N* = 18, for those whose self-categorized self as doer identification was inconsistent across time. In contrast, there was no significant correlation between strength of self as doer identification and anxiety for those whose self-categorized active identifications were consistent: *r* = 0.01, *p* = 0.73, *N* = 14.

**FIGURE 2:**
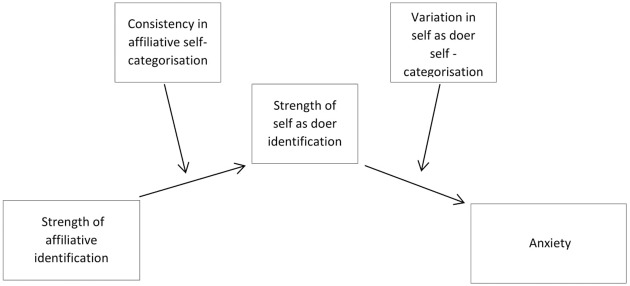
**Moderated mediation in the relationship between strength of off illative identification and anxiety via strength of self as doer identification, moderated by stability of affiliative and self as doer self-categorisations**.

Moderated mediation analysis was conducted [using PROCESS model 21 (Hayes, 2013) and SPSS v.21]. Confidence intervals were set at 99%. The model controlled for gender and cause of injury (because as illustrated in the associations between identities, demographics, and anxiety section above, gender and cause of injury were significantly related to strength of identification in the preliminary analysis). There was a significant conditional indirect effect of affiliative identification on anxiety when there was consistency in affiliative self-categorisation and inconsistency in self as doer self-categorisation *B* = -0.29, *SE* = 0.10, 99%CI [-0.58, -0.02] (see **Figure 2**). In contrast, where there was consistency in both affiliative self-categorisation and self as doer self-categorisation the indirect effect of strength of affiliative identification on anxiety was not significant: *B* = -0.03, *SE* = 0.14, 99%CI [-0.43, 0.32]. Amongst those for whom there was inconsistency in affiliative self-categorisation and consistency in self as doer self-categorisation the indirect effect of strength of affiliative identification on anxiety was not significant: *B* = 0.01, *SE* = 0.13, 99%CI [-0.50, 0.46]. Similarly, with inconsistency in both affiliative and self as doer self-categorisations the indirect effect of strength of affiliative identification on anxiety was not significant: *B* = 0.04, *SE* = 0.27, 99% CI [-0.716, 1.16].

The direct effect of strength of affiliative identification on anxiety was not significant *B* = 0.21, *SE* = 0.09, 99% CI [-0.05, 0.47].

PROCESS does not generate standardized coefficient figures where covariates are included in the model. The overall model fit was *R*^2^= 0.47, *p* = 0.02. Results indicate a significant conditional indirect effect of affiliative identification on anxiety when there was consistency in affiliative self-categorisation and inconsistency in self as doer self-categorisation *B* = -0.29, *SE* = 0.10, 99% CI [-0.58, -0.02].

## Discussion

These results were a surprise to us. We had expected to find consistency of identification in terms of relationships between both affiliative and ‘self as doer’ identity types and anxiety was the significant pathway. Instead, we found that the significant identity combination was consistency of identification regarding affiliative identity and inconsistency of identification regarding ‘self as doer.’ How can we explain these findings?

Beginning with affiliative identities: results of the present study indicate that identification with the groups that ABI survivors belong to, groups such as family, constitute a base from which individuals can construct self as doer identities. This finding is consistent with the social identity approach advocated by Haslam et al. (2009) that builds on the self-categorisation theorizing of Turner (1982) who suggested that social identities are a resource that makes group behavior possible. The applied social identity approach recently propounded by Haslam (2014) suggests that when people are severely incapacitated, in situations such as that pertaining after ABI, the groups that they belong to constitute a resource that can contribute to sustaining them. Family is perhaps the most obvious example but clubs, communities and social circles also offer resources with the capacity to contribute perceptions of consistency and maintain individuals in times of acute stressors such as ABI. The results of the present study are supportive of the idea that belonging to groups offers a basis from which individuals can engage with meaningful functional activities that then become internalized as identities (Douglas, 2012). Strongly identifying with the groups they belong to and being consistent in that self-categorisation were related to lower levels of anxiety amongst those who participated in this study. This much was predicted.

However, the next finding was not predicted. The indirect pathway from affiliative identification to anxiety was significant for those who reported different self as doer identities across times 1 and 2. It was *not* significant for those who were consistent across time points in their self as doer self-categorisations. We had predicted that this mediated aspect of the relationship between strength of self as doer identification and anxiety would be moderated such that the relationship would be significant for those with consistent self as doer self-categorisations. This is not what was found. The data indicated that the relationship between strength of self as doer identification was significant for those whose self-categorisations were inconsistent.

One way to make sense of this finding is to reflect on our language. We thought we were observing ‘inconsistency’ – perhaps what we were observing was ‘flexibility’ of identification. Could it be that those with the support provided by consistent affiliative identification had a more flexible range of ‘self as doer’ identifications available to them and were thus ‘better’ in terms of outcome? This may be because the use of alternative self as doer identifications evidences adjustment and flexibility in self-categorisation. Strength of self as doer self-categorisation mattered, in this instance, only for those participants who reported different self as doer identities at each time point. For this group of people there was a significant negative relationship between self as doer identification and anxiety: the stronger their self as doer identity the less anxious they were likely to be. We argue that an explanation for this may be as follows: in parallel with understanding the importance of collective identification across time, Haslam et al. (2008) emphasized the significance of multiple identifications for psychological well-being after stroke. Haslam et al. (2008) have shown that belonging to multiple social groups following stroke is a better predictor of psychological well-being than reported cognitive difficulties or than pre-injury group memberships. Iyer et al. (2009) presented findings that show compatibility between old and new identities and that having multiple group memberships increases the likelihood that individuals will identify with new groups at a time of major upheaval or change in their lives. Importantly, Iyer et al. (2009) also demonstrated that identification with a new group can buffer individuals from the negative effects of change on psychological well-being. Stability and multiplicity regarding identity are thus both relatively well-established as important to well-being after ABI. It seems that the findings of this study may reflect the importance of both and that the results go some way toward accounting for stability and multiplicity through distinct identity sub-types. Stability was required in order for affiliative identities to exert a significant effect and variability in self as doer identities was required for them to exert a significant effect.

Haslam (2014) argues that groups and social identities matter. In the context of post-acute ABI survivors, the results of this study support Haslam’s position. Gracey and Ownsworth (2012) focused on processes involved in survivor’s capacity to internalize an adaptive and coherent post-injury identity and found that engagement in meaningful activities with which individuals identify contributes to emotional adjustment. Again, the present study supports the position advocated by Gracey and Ownsworth. What the present study adds to these studies is the understanding that identifications with different identity sub-types, and the stability of these identifications, are a significant factor requiring consideration. It is our hope that the findings presented herein might serve to inform clinical practice and stimulate further research. In this regard, some additional suggestions advanced by Haslam (2014) are especially salient. First, the power of social identity can be unlocked by working with rather than against it. We suggest that the concepts of affiliative and self as doer identities might offer the basis for interventions designed around, and working with, the understandings that people have of themselves following ABI. We have provided evidence that continuity of belonging and multiplicity of doing identities are associated with lower levels of anxiety and we hope that this finding will constitute a basis for future research both by ourselves and others. Haslam (2014) also argues that social identities must be made to matter. This suggestion raises intriguing possibilities in the context of ABI rehabilitation. Haslam (2014) suggests that it is possible to work with individuals in such a fashion as to transform abstract notions of ‘us’ into lived experience. Participation in meaningful activities, as suggested by Gracey and Ownsworth (2012), and others (Gracey et al., 2008; Douglas, 2012) may be one avenue via which this end can be achieved. Provision of structured, meaningful activities in groups organized by rehabilitation service providers is one practical example of the type of facilitated identification with activity advocated by researchers including Douglas (2012) and Gracey and Ownsworth (2012) and supported by the results of the present study. Again, we hope that the distinction between identity sub-types that has been evidenced in this study might offer a point of embarkation for the development of practical interventions that might further develop and apply this insight.

### Limitations

The size of the sample presented in this study small and it is difficult to generalize from such a limited and apparently heterogeneous sample. The influence of cognitive impairment was not taken into account and it is possible that a lack of insight impacted the results in ways we have not considered. Another important factor that should be attended to in future research is disability. Additionally, were predominantly white, male, and Irish. This raises the question of culture – are cultural factors playing a ‘hidden hand’ in the dynamics that correlational analysis identified?

## Conclusion

In sum, our results suggest that in the context of post-acute neurorehabilitation social identity theorizing, a social neuropsychological approach (Haslam et al., 2008; Gracey and Ownsworth, 2012) is ‘good.’ It offers predictive and practical utility. Haslam (2014), one of the principal drivers of contemporary social identity theorizing, quotes Lewin’s (1951) dictum that ‘there is nothing as practical as a good theory.’ If Lewin, and Haslam, are correct (and we suggest that they are), it is incumbent upon clinicians and researchers alike to explore innovative ways of making the type of theoretically informed social neuropsychological approach advocated by Haslam et al. (2008), Gracey and Ownsworth (2012), and others practical through application. Morse (1997) suggest that identity discontinuity and threat to integrity is central to the experience of illness. We suggest that identifying with the group that ABI survivors belong to and participation in meaningful activities can contribute to a sense of shared social identity, the experience of personal continuity, integrity and wholeness in survivors of ABI. It is our hope the research presented herein might contribute to this end.

## Author Contributions

All authors listed have made substantial, direct and intellectual contributions to the work, and approved it for publication.

## Conflict of Interest Statement

The authors declare that the research was conducted in the absence of any commercial or financial relationships that could be construed as a potential conflict of interest.

## Footnotes

1 The specific questions used to elicit affiliative and ‘self as doer’ self-categorisations are provided in the Section “Materials and Methods.”

2 ‘Other causes’ is essentially TBI participants + 2 participants with hypoxia and 1 whose cause of injury is unknown.
