# Supplementary material for: A Longitudinal Study of Relationships between Identity Continuity and Anxiety Following Brain Injury
Source: Front Psychol. 2017 May 12;8:648. doi: 10.3389/fpsyg.2017.00648 (PMC5427107; doi:10.3389/fpsyg.2017.00648)
Supplement: Supplementary file 1 [file Data_Sheet_1.docx]

**Appendix 1.**

**Self-categorised ‘self as doer’ identities at T1 and T2**

| **Time 1 self as doer identity** | **Time 2 self as doer identity** | **Continuity Code** |
| --- | --- | --- |
| following Munster rugby  child care  going to matches  thinking  farming  Mechanical work  art  coffee with friends  listening to music  theatre/cinema  baking  parenting  fitness  fixing / being handy  parenting  photography  music & guitar  music  gym/pool/sauna  walking  farming  mother  walking  walking  following GAA  music  greyhounds  computers  going up the town  cooking and baking  dressing/clothes  bird breeding | following rugby  home life  sport  thinking  farming  Mechanical work / fix tractors  art  socialising  music  cinema  baking  family  running  handyman  praying /AA  service provider activities  caring for grandmother  following sport  Poker  getting out  being out and about  collecting  being a dad  socialising  DIY group  college  walking  getting hair done  exercises  shopping  spiritualism  parenting | 0  0  0  0  0  0  0  0  0  0  0  0  0  0  1  1  1  1  1  1  1  1  1  1  1  1  1  1  1  1  1  1 |

**Appendix 2:**

**Self-categorised affiliative identities at T1 and T2**

| **Time 1 affiliative identity** | **Time 2 affiliative identity** | **Continuity Code** |
| --- | --- | --- |
| family  married  friends  friends  family  family  family  my kids  family  friends  family  family  family  my family  family  family  family  family  friends  family  family  family  family  family  Rehab group  friends  people in bookies  friends  my partner  family  humankind  family | family  family  friends  friends  family  family  family  family  family  family  family  family  family  Rehab group  family  husband  family  family  friends  family  family  family  family  family  family  people  family  Rehab group  friends  GAA  Rehab group  Rehab group | .00  .00  .00  .00  .00  .00  .00  .00  .00  .00  .00  .00  .00  1.00  .00  .00  .00  .00  .00  .00  .00  .00  .00  .00  1.00  1.00  1.00  1.00  1.00  1.00  1.00  1.00 |
